# Cribriform Tumor Burden in Grade Group 4 Prostate Cancer: A Quantitative Threshold Predicting Lymphovascular Invasion and Metastasis

**DOI:** 10.3390/jcm15062303

**Published:** 2026-03-18

**Authors:** Ilkay Tosun, Onur Sahin, Eyup Veli Kucuk

**Affiliations:** 1 Department of Pathology, Umraniye Training and Research Hospital, University of Health Sciences, 34764 Istanbul, Turkey; sahinonur.md@gmail.com; 2 Department of Urology, Umraniye Training and Research Hospital, University of Health Sciences, 34764 Istanbul, Turkey; eyupveli@hotmail.com

**Keywords:** prostate cancer, Grade Group 4, cribriform tumor burden, lymphovascular invasion, prognosis

## Abstract

**Background/Objectives**: Although the presence and diameter of the cribriform pattern (CP) are established prognostic factors in prostate cancer (PCa), the clinical impact of quantitative cribriform tumor burden (CTB) remains poorly characterized. This study aimed to evaluate the association between CTB and clinicopathological outcomes in Grade Group 4 PCa with large cribriform morphology (LC-GG4). **Methods**: We retrospectively analyzed patients with pure GG4 prostate cancer exhibiting ≥1 large cribriform gland (>0.25 mm) at radical prostatectomy. CTB was assessed as the percentage of cribriform architecture relative to the total tumor area. Following clinicopathological correlation, receiver operating characteristic (ROC) analysis determined the optimal CTB threshold for predicting lymphovascular invasion (LVI). Distant Metastasis-free survival (dMFS) and biochemical recurrence-free survival (BCRFS) were evaluated using the Kaplan–Meier and log-rank tests. **Results**: In 43 patients with LC-GG4, extraprostatic extension was present in 100% of cases. The median CTB was 30.0% (IQR: 15.0–60.0%). A CTB threshold of ≥25% was optimally associated with LVI (area under the curve [AUC]: 0.801, *p* = 0.002). High-CTB (≥25%) was strongly correlated with LVI (*p* = 0.002) and intraductal carcinoma (*p* = 0.004) and was independently associated with LVI in multivariate analysis (OR: 1.054; *p* = 0.006). Furthermore, high-CTB patients demonstrated significantly shorter mean dMFS (84.9 vs. 113.1 months; *p* = 0.042), with no significant difference observed for BCRFS. **Conclusions**: In LC-GG4 prostate cancer, CTB is a critical determinant of clinical aggressiveness. A quantitative threshold of ≥25% was independently associated with LVI and early metastatic progression. Quantifying CTB, rather than relying on simple binary assessment, provides superior risk stratification.

## 1. Introduction

The presence of cribriform pattern (CP) in prostate adenocarcinoma (PCa) is recognized as an independent adverse prognostic factor associated with unfavorable clinical outcomes, including biochemical recurrence (BCR) and distant metastasis [1,2]. Although currently categorized under Gleason pattern 4, accumulating evidence suggests that CP represents a distinct biological entity exhibiting significantly more aggressive behavior than other pattern 4 subtypes, such as poorly formed or fused glands [3,4]. Consequently, precise identification and standardized reporting of this morphology are essential for risk stratification in patients with PCa.

Although reporting the presence of CP in risk assessments is important, the underlying histomorphological features, particularly large cribriform glands, provide more detailed information for predicting clinical outcomes [1,5]. However, the prognostic significance of quantifying the percentage or extent of CP, defined as cribriform tumor burden (CTB), has not been fully elucidated [5,6]. The prognostic significance of CP and histomorphological features has been investigated in both low-risk (Grade group (GG) 1–2) [7] and high-risk (GG5) groups; however, there are few studies specifically on pure GG4 patients, who have a heterogeneous clinical presentation [8].

Therefore, this study aimed to evaluate the association between CTB and adverse clinicopathological outcomes in GG4 PCa with large cribriform pattern (LC-GG4). Such an assessment may facilitate the development of refined risk stratification models and tailored surveillance strategies for the clinically heterogeneous population of GG4 patients.

## 2. Materials and Methods

This retrospective study was conducted at the University of Health Sciences, Umraniye Training and Research Hospital, Department of Pathology, Istanbul, Turkey, in accordance with the Declaration of Helsinki. The study protocol was approved by the Institutional Ethics Committee (B.10.1.TKH.4.34.H.GP.0.01/262).

A retrospective review of the institutional database identified a total of 1871 patients who underwent robot-assisted radical prostatectomy (RP) between 2015 and 2024. Patients who had undergone neoadjuvant androgen deprivation therapy or radiation therapy, and those with inadequate clinical follow-up, were excluded. Among the eligible cohort, 57 patients with pure GG4 disease without a tertiary Gleason pattern 5 component were identified. The Gleason score distribution for these 57 GG4 cases was as follows: 3 + 5 (*n* = 1), 4 + 4 (*n* = 54), and 5 + 3 (*n* = 2).

All slides were independently reviewed by two pathologists (I.T. and O.S.). Histopathological review of the 57 pure GG4 cases revealed that 8 patients had no cribriform pattern, whereas 49 patients (all with Gleason score 4 + 4) exhibited cribriform morphology. Cases with only small cribriform glands (≤0.25 mm in their longest axis; *n* = 6) were subsequently excluded. Ultimately, the study cohort was restricted to 43 patients who met the strict inclusion criterion of exhibiting at least one ‘large’ cribriform gland, defined as having a longest axis >0.25 mm. In cases of discrepancy regarding the initial evaluations, a consensus was reached using a multi-headed microscope.

For each case, CTB was determined through a semi-quantitative assessment, involving a review of all tumor-involved slides to estimate the percentage of the total tumor area occupied by CP (encompassing both small and large glands). Total tumor volume (TTV) was determined through visual estimation (eyeballing) of the tumor area across all involved slides. CTB was recorded as a continuous variable (%).

Given the shared aggressive biology and morphological overlap, CTB was defined as the total cribriform burden, including both invasive cribriform carcinoma and intraductal carcinoma of the prostate (IDC-P) components. To distinguish between the two entities, initial evaluations were based on routine hematoxylin and eosin (H&E) morphology. In all cases with suspected IDC-P, the diagnosis and the presence of a preserved basal cell layer were confirmed using p63 immunohistochemistry.

Additional histopathological parameters, including extraprostatic extension (EPE), seminal vesicle invasion (SVI), lymphovascular invasion (LVI), and surgical margin status, were documented.

The primary endpoints were distant metastasis-free survival (dMFS) and biochemical recurrence-free survival (BCRFS). dMFS was calculated from the date of surgery to the radiological or pathological confirmation of distant metastasis, independent of regional lymph node status at the time of surgery, or to the last follow-up. BCRFS was defined as the interval between surgery and biochemical recurrence (two consecutive PSA values > 0.2 ng/mL) or the last follow-up.

Statistical analyses were performed using IBM SPSS Statistics (version 27.0; IBM Corp., Armonk, NY, USA). Continuous variables were expressed as mean ± standard deviation (SD), mean ± standard error (SE), or median (interquartile range [IQR]), as appropriate, while categorical variables were presented as frequencies and percentages. Associations between categorical variables were evaluated using Fisher’s exact test. The Mann–Whitney U test was employed to compare continuous variables across groups. Receiver operating characteristic (ROC) curve analysis and the Youden index were utilized to determine the optimal cutoff value for CTB in predicting adverse outcomes. Survival curves were generated using the Kaplan–Meier method and compared using the log-rank test. Statistical significance was set at *p* < 0.05.

## 3. Results

### 3.1. Clinicopathological Characteristics

**Figure 1:**
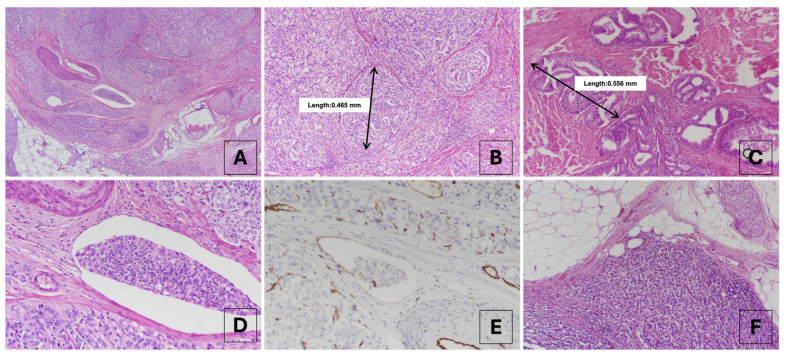
Histopathological characteristics of Grade Group 4 prostate cancer exhibiting large cribriform morphology. (**A**–**C**) Representative photomicrographs demonstrate large invasive cribriform carcinoma (>0.25 mm). Note the expansile growth pattern, back-to-back arrangement, and characteristic multiple punched-out lumina with loss of cell polarity (H&E, ×40, ×100, and ×100, respectively). (**D**) Identification of lymphovascular invasion (LVI). A cohesive cluster of cribriform tumor cells was observed within a vascular space adjacent to the tumor (H&E, ×200). (**E**) CD31 immunohistochemistry highlights the endothelial lining of the vessel, confirming the presence of tumor emboli within the vascular lumen (IHC, ×200). (**F**) Extraprostatic extension (EPE) reveals tumor glands extending beyond the prostatic capsule and infiltrating the periprostatic adipose tissue (H&E, ×40).

Forty-three patients who met the inclusion criteria for LC-GG4 were included in the final analysis. The mean age was 64.2 ± 4.9 years, and the median preoperative PSA level was 13.5 ng/mL (IQR: 7.7–20.0 ng/mL). Histopathological examination revealed that all tumors were Gleason score 4 + 4 = 8 and characterized by large cribriform glands (>0.25 mm) exhibiting expansile growth patterns (Figure 1A–C). LVI was identified in 13 (30.2%) patients (Figure 1D), with equivocal foci confirmed by CD31 immunohistochemistry (Figure 1E). Notably, EPE was present in all cases (100%), characterized by tumor glands infiltrating the periprostatic adipose tissue (Figure 1F). SVI was observed in 22 (51.2%) patients, and IDC-P accompanied the invasive component in 5 (11.6%) cases. The detailed clinicopathological parameters are summarized in Table 1.

### 3.2. Association Between Cribriform Tumor Burden and Histopathological Findings

**Figure 2:**
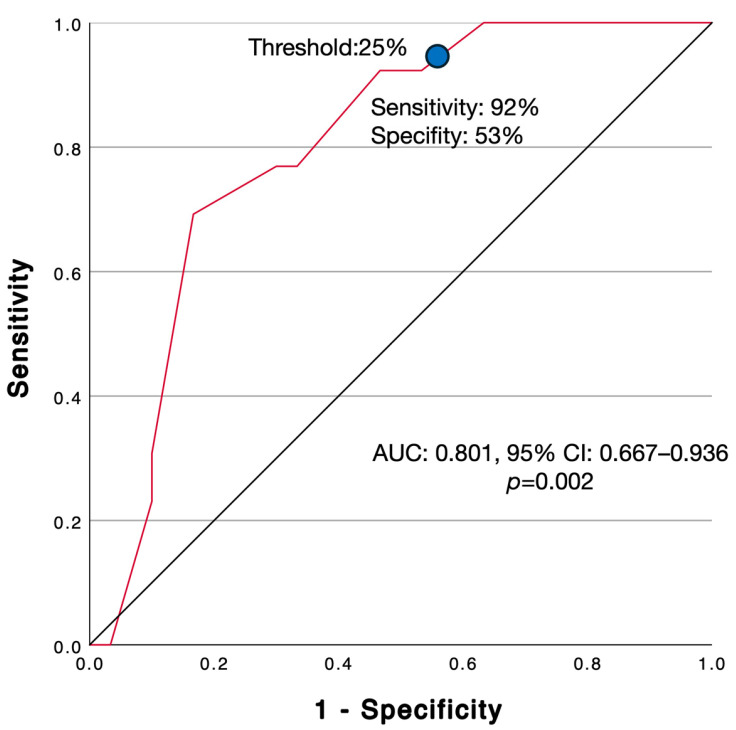
Receiver Operating Characteristic (ROC) curve analysis evaluating the predictive value of Cribriform Tumor Burden (CTB) for Lymphovascular Invasion (LVI). The optimal threshold was ≥25% (indicated by the blue dot), with a sensitivity of 92% and specificity of 53% (AUC: 0.801; 95% CI: 0.667–0.936; *p* = 0.002).

The median CTB was 30.0% (IQR: 15.0–60.0%). Initial comparative analysis revealed a statistically significant relationship between CTB (%) and LVI (*p* = 0.002). ROC curve analysis was performed to identify the optimal prognostic threshold for predicting LVI. The analysis confirmed that CTB was significantly associated with LVI (AUC: 0.801, 95% CI: 0.667–0.936; *p* = 0.002) (Figure 2). The Youden index identified an optimal cut-off of ≥25% (sensitivity: 92.3%; specificity: 53.3%). Applying this cut-off value, patients were stratified into two groups: 15 (34.9%) with low-CTB (<25%) and 28 (65.1%) with high-CTB (≥25%). As detailed in Table 1, the high-CTB phenotype was significantly associated with aggressive characteristics, exhibiting a higher prevalence of both LVI (*p* = 0.002) and IDC-P (*p* = 0.004). To ensure the robustness of this finding and exclude cut-off bias, a sensitivity analysis was performed using the unbiased median CTB value (30%) as the threshold. The significant association between CTB and LVI persisted (*p* = 0.006), confirming that the correlation is biologically robust and independent of the selected cut-off. Furthermore, to confirm that this association was driven by invasive cribriform architecture rather than the intraductal component, an additional sensitivity analysis was performed excluding the 5 patients with IDC-P. In the remaining pure invasive LC-GG4 cohort (*n* = 38), the significant association between high CTB (≥25%) and LVI persisted (Fisher’s Exact Test, *p* = 0.026).

Notably, the total tumor volume (TTV) was significantly higher in patients with LVI (*p* = 0.033). To evaluate whether CTB was associated with LVI independently of TTV, binary logistic regression analysis was performed. Univariate analysis revealed that CTB was significantly associated with LVI (OR: 1.057, 95% CI: 1.019–1.096; *p* = 0.003), while TTV did not reach statistical significance (OR: 1.041, 95% CI: 0.993–1.092; *p* = 0.096). In a multivariate model adjusted for TTV, CTB was significantly associated with LVI (OR: 1.054, 95% CI: 1.015–1.094; *p* = 0.006) (Table 2).

### 3.3. Survival Analysis

**Figure 3:**
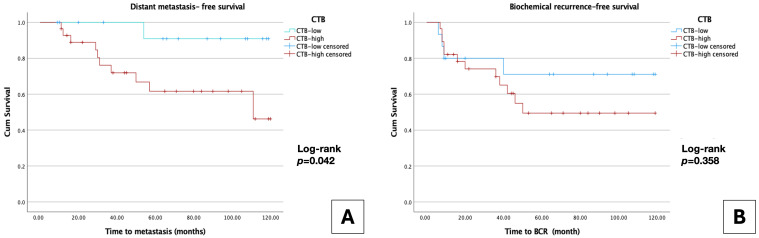
Kaplan–Meier survival analysis stratified by Cribriform Tumor Burden (CTB). (**A**) Metastasis-free survival (dMFS): Patients in the high-CTB group exhibited significantly shorter dMFS than those in the low-CTB group (log-rank *p* = 0.042). (**B**) Biochemical recurrence-free survival (BCRFS): Although an earlier recurrence trend was observed in the high-CTB group, the difference did not reach statistical significance (log-rank *p* = 0.358).

Kaplan–Meier analysis demonstrated a significant prognostic impact of CTB on dMFS. Patients in the High-CTB (≥25%) group exhibited a significantly shorter mean dMFS of 84.9 ± 8.9 months than those in the Low-CTB (<25%) group (113.1 ± 5.6 months; log-rank *p* = 0.042) (Figure 3A). Regarding BCRFS, although the High-CTB group exhibited a numerically shorter mean recurrence-free time than the Low-CTB group (72.3 ± 9.8 months vs. 89.7 ± 12.5 months), this difference was not statistically significant (log-rank *p* = 0.358) (Figure 3B).

## 4. Discussion

In this study, we investigated the prognostic utility of quantitative CTB in a homogeneous cohort of LC-GG4 PCa. Our findings indicate that CTB ≥ 25% was independently associated with LVI and was significantly associated with reduced dMFS. These results suggest that a “critical threshold” of burden is required to manifest the most aggressive metastatic phenotype in GG4 disease.

Complementing our inclusive approach to IDC-P, a defining feature of this study is the exclusive focus on “large” cribriform morphology (diameter > 0.25 mm). While earlier investigations often analyzed CP as a single entity regardless of gland size, recent data indicate that “size matters” in risk stratification. Chan et al. rigorously demonstrated that cribriform glands exceeding a diameter of 0.25 mm are the true drivers of adverse pathology and biochemical failure, whereas smaller cribriform variants often lack independent prognostic significance in multivariable models [5,9]. By restricting our cohort to this biologically distinct “large” phenotype, we observed a more uniformly aggressive disease profile—exemplified by the invariant (100%) presence of extraprostatic extension—compared to literature cohorts that pooled small and large variants.

Our results corroborate a rapidly emerging paradigm in uropathology suggesting that the volume of CP drives clinical outcomes. While earlier guidelines focused on the binary presence of the pattern, recent data support a dose-dependent risk stratification. However, the optimal threshold remains a subject of debate and varies largely by the risk profile of the cohort studied. For instance, Tekin et al. reported that in Gleason Score 7 tumors, invasive cribriform glands occupying >10% of the tumor area were significantly associated with BCR [10]. Similarly, Chen et al. emphasized that the percentage of CP is a superior prognosticator compared to its simple presence, particularly in mixed GGs [11]. In more advanced disease, higher thresholds appear to be necessary to stratify risk; Okubo et al. identified a cutoff of ~44.5% positive cores as critical for metastatic progression in castration-sensitive PCa [12]. Shimodaira et al., focusing on a cohort of GG4 similar to ours, also found that risk escalates in parallel with tumor burden [8]. In this context, our identified threshold of ≥25% in pure GG4 represents a critical intermediate inflection point. It suggests that in high-grade disease, a “minor” cribriform component may already be biologically active, but a substantial burden (one-quarter of the tumor volume) is required to drive the phenotype towards distant metastasis rather than local recurrence.

Beyond survival outcomes, the association between quantitative CTB and adverse histopathological parameters reinforces the concept that tumor volume drives local aggressiveness. In the literature, particularly within Gleason Score 7 cohorts, studies by Chen et al. and Tekin et al. have consistently demonstrated that increased cribriform percentage correlates with EPE and advanced pathological stage [10,11]. However, the invariant presence of EPE (100%) in our LC-GG4 PCa cohort suggests that this specific morphology serves as a definitive marker of locally advanced disease (pT3a), independent of the burden percentage. Conversely, regarding LVI, the literature findings are conflicting; while Tekin et al. reported a strong association [10], Chen et al. found no significant correlation in intermediate-risk disease [11]. Our study resolves this ambiguity for high-grade disease, demonstrating that LVI risk becomes statistically significant when CTB exceeds a threshold of 25%. This indicates that increasing CTB facilitates not only stromal invasion but also vascular entry. Furthermore, the significant association between high-CTB and IDC-P coexistence supports the presence of a “high-density” aggressive phenotype characterized by simultaneous invasive and intraductal spread.

Invasive cribriform pattern and IDC-P exhibit substantial morphological overlap, rendering definitive distinction challenging on H&E-stained sections [5]. The current literature emphasizes that both entities share comparable genomic instability profiles and confer similar adverse prognostic outcomes regarding BCR, distant metastasis, and disease-specific mortality [13]. Given these molecular and clinical parallels, we incorporated IDC-P foci within the spectrum of cribriform morphology, and IDC-P components were included in the quantitative calculation of CTB.

Current literature consistently associates increased cribriform percentage with lower BCRFS, particularly in intermediate-risk cohorts. Studies by Tekin et al. and Leo et al. established a quantitative threshold of >10% as an independent predictor of BCR in GG2 and GG3 patients [10,14]. Similarly, Chen et al. reported that a cribriform percentage >20% in Gleason Score 7 tumors was significantly associated with earlier biochemical failure [11]. In contrast, our analysis of a pure GG4 cohort did not demonstrate a statistically significant difference in BCRFS between High-CTB and Low-CTB groups; however, a trend towards earlier recurrence was observed in high-burden cases. We attribute this discrepancy to a “ceiling effect” inherent to our study design. Unlike the heterogeneous intermediate-risk populations cited in the literature, our cohort consisted exclusively of pure LC-GG4 disease with invariant EPE (100%). Within this uniformly high-risk population, the baseline probability of biochemical failure is elevated regardless of burden, diminishing the discriminatory power of CTB for this specific endpoint. Furthermore, the relatively low overall BCR rate (37%) in this high-risk, EPE-positive cohort is largely attributable to the administration of early adjuvant therapies (androgen deprivation therapy and/or radiotherapy), which are standard of care in our institution for patients with such adverse pathological features. This finding aligns with a study suggesting that the prognostic impact of cribriform architecture is most pronounced in lower-grade disease and may plateau in advanced risk groups [7].

A striking finding of our study is the significant association between a high CTB (≥25%) and reduced dMFS, identifying cribriform burden as a driver of systemic rather than local progression. Only one patient in the low-CTB group developed metastasis, whereas ten patients in the high-CTB group progressed to metastatic disease. This corroborates the work of Kweldam et al., who identified CPs as independent predictors of distant metastasis and disease-specific mortality [15]. Furthermore, Seyrek et al. demonstrated that grading systems incorporating cribriform burden (cGrade) outperform standard grading in predicting dMFS (C-index 0.834) [6]. Biologically, this suggests that as the cribriform burden exceeds a critical threshold—identified here as ≥25% for GG4 disease—the tumor phenotype shifts from locally aggressive to systemically potent. This is supported by Elfandy et al., who showed that invasive cribriform carcinoma shares molecular similarities with metastatic castration-resistant prostate cancer, including enriched *MYC* and *mTORC1* pathway activity [16]. Therefore, while a 10–20% threshold may suffice to predict BCR in intermediate-risk disease, our findings indicate that a higher burden (≥25%) is required to manifest the metastatic phenotype in high-grade disease.

The present study has potential implications for the management of high-grade PCa, pending validation in larger cohorts. First, the ubiquitous presence of EPE observed in our study indicates that large cribriform morphology may serve as an indicator of locally advanced disease. Given that biopsy sampling often underestimates the true extent of cribriform architecture, as noted by Osiecki et al., the identification of large cribriform glands in preoperative specimens warrants careful surgical planning, particularly when considering nerve-sparing procedures [17]. Second, the identification of a ≥25% threshold for metastatic risk suggests that quantifying CTB could provide valuable prognostic information beyond standard grading. Patients with GG4 disease and high CTB (≥25%) appear to face a heightened risk of systemic progression. Consequently, in addition to standard protocols, these patients might benefit from closer postoperative monitoring and consideration for molecular risk assessment to guide multimodal management strategies, consistent with the aggressive molecular features described in the recent literature [18].

The current study has several limitations. First, the retrospective design and relatively small sample size limit the statistical power of certain sub-analyses, such as the ceiling effect observed in BCRFS. The low number of metastasis events (*n* = 11) precluded the use of multivariable Cox proportional hazards modeling to avoid overfitting. Second, our ROC-derived threshold of ≥25% lacks internal validation (e.g., bootstrapping) and might be sensitive to small shifts in event distribution, necessitating external validation. Third, the semi-quantitative visual estimation of CTB introduces potential inter-observer variability, although major discrepancies were resolved via consensus. Fourth, although we specifically evaluated well-formed cribriform areas to minimize artifacts, measuring the longest axis remains susceptible to the plane of sectioning. Unlike previous studies that used the shortest axis, our approach may potentially lead to a slight overestimation of gland size. Finally, because our inclusion criteria specifically selected for “large” cribriform glands, our final cohort exclusively consisted of pT3 cases. Therefore, these findings cannot be generalized to organ-confined (pT2) disease without further study.

## 5. Conclusions

GG4 is not a uniform entity. Within this high-risk population, the extent of large CP is a critical determinant of tumor aggressiveness. We identified a quantitative threshold of ≥25% CTB that was independently associated with LVI and distant metastasis. Furthermore, the universal presence of EPE indicates that large cribriform morphology is a robust marker for locally advanced disease. We recommend routine reporting of CTB in pathology reports to refine risk stratification and guide personalized management.

## Figures and Tables

**Table 1:** Association of cribriform tumor burden (CTB) with clinicopathological characteristics.

	*Low-CTB* (<25%) (*n* = 15)	*High-CTB* (≥25%) (*n* = 28)	*p*
**Lymphovascular invasion**, *n* (%)	1 (6.7%)	12 (42.9%)	* 0.002 ^a^
**Intraductal carcinoma**, *n* (%)	0 (0.0%)	5 (17.9%)	* 0.004 ^a^
**Age (years)**, *mean* ± *SD*	64.2 ± 5.2	64.2 ± 4.8	0.842 ^b^
**Preoperative PSA (ng/mL)**, *Median* (*IQR*)	10.6 (4.7–22.5)	14.0 (7.4–36.5)	0.215 ^b^
**Tumor volume (%)**, *Median* (*IQR*)	18.0 (5.0–40.0)	25.0 (13.5–29.5)	0.096 ^b^
**Pathological T Stage**, *n* (%)			0.138 ^a^
*pT3a*	9 (60.0%)	12 (42.9%)	
*pT3b*	6 (40.0%)	16 (57.1%)	
**Pathological N Stage**, *n* (%)			0.910 ^c^
***pN0***	9 (60.0%)	15 (53.6%)	
***pN1***	3 (20.0%)	8 (28.6%)	
***pNx***	3 (20.0%)	5 (17.9%)	
**Positive Margin (R1)**, *n* (%)	11 (73.3%)	21 (75.0%)	0.640 ^a^
**Ductal adenocarcinoma component**, *n* (%)	3 (20.0%)	6 (21.4%)	0.367 ^a^
**Multifocality**, *n* (%)	3 (20.0%)	5 (17.9%)	0.739 ^a^
**Clinical outcomes**, *n* (%)			
*Metastasis*	1 (6.7%)	10 (35.7%)	* 0.042 ^d^
*Biochemical recurrence*	4 (26.7%)	12 (42.9%)	0.358 ^d^
**Follow-up (months), ***mean* ± *SE*			
*Time to metastasis*	113.1 ± 5.6	84.9 ± 8.9	
*Time to biochemical recurrence*	89.7 ± 12.5	72.3 ± 9.8	

^a^ Fisher’s Exact Test; ^b^ Mann–Whitney U Test; ^c^ Fisher-Freeman-Halton Exact Test; ^d^ Log-Rank Test; *, Statistically significant (*p* < 0.05).

**Table 2:** Univariate and multivariate logistic regression analysis for predictors of lymphovascular Invasion (LVI).

	*Univariate*		*Multivariate*	
	*OR* (95% *CI*)	*p*	*OR* (95% *CI*)	*p*
**Cribriform tumor burden**	1.057 (1.019–1.096)	0.003	1.054 (1.015–1.094)	0.006 *
**Total tumor volume (%)**	1.041 (0.993–1.092)	0.096	1.028 (0.980–1.079)	0.262

*, Statistically significant (*p* < 0.05); Model statistics: χ^2^ = 12.459, *p* = 0.002; Nagelkerke R^2^ = 0.356; Hosmer-Lemeshow *p* = 0.375.

## Data Availability

The original contributions presented in this study are included in the article. Further inquiries can be directed to the corresponding author.

## Abbreviations

The following abbreviations are used in this manuscript:

AUC	Area Under the Curve
BCR	Biochemical Recurrence
BCRFS	Biochemical Recurrence-Free Survival
CI	Confidence Interval
CP	Cribriform Pattern
CTB	Cribriform Tumor Burden
EPE	Extraprostatic Extension
GG	Grade Group
IDC-P	Intraductal Carcinoma of the Prostate
IQR	Interquartile Range
LC-GG4	Grade Group 4 Prostate Cancer with Large Cribriform Pattern
LVI	Lymphovascular Invasion
dMFS	Distant Metastasis-Free Survival
OR	Odds Ratio
PCa	Prostate Adenocarcinoma
PSA	Prostate-Specific Antigen
ROC	Receiver Operating Characteristic
RP	Radical Prostatectomy
SD	Standard Deviation
SE	Standard Error
SVI	Seminal Vesicle Invasion
TTV	Total Tumor Volume
